# Supplementary figures and images for: A familial missense variant in the Alzheimer’s disease gene SORL1 impairs its maturation and endosomal sorting
Source: Acta Neuropathol. 2024 Jan 20;147(1):20. doi: 10.1007/s00401-023-02670-1 (PMC10799806; doi:10.1007/s00401-023-02670-1)

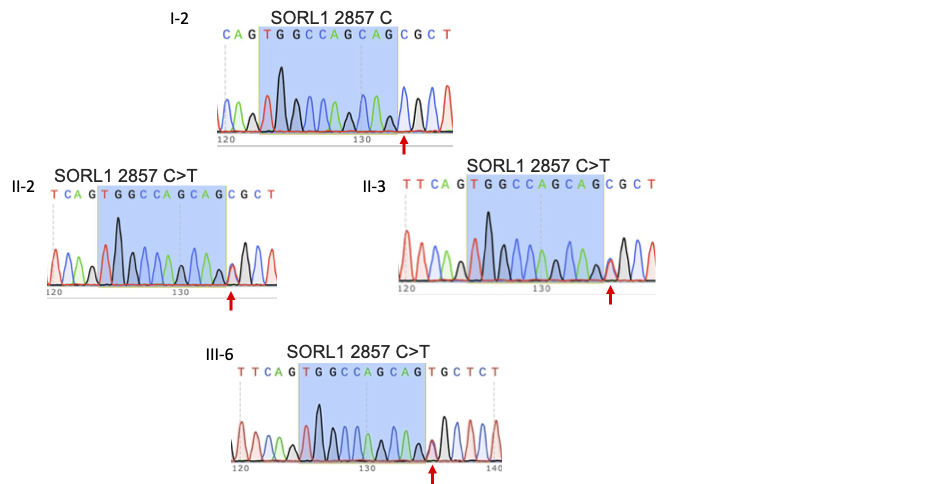

Supplement: Supplementary file 1 — Supplementary file1. Sanger sequencing results for I-2, II-2, II-3, III-6. I-2 is C/C at SORL1 2857. II-2, II-3, III-6 are C/T at SORL1 2857. (TIF 1779 KB) [file 401_2023_2670_MOESM1_ESM.tiff]
